# TMAO Induced Kidney Aging by Activating ZBP1-Mediated Necroptosis

**DOI:** 10.33549/physiolres.935612

**Published:** 2026-02-01

**Authors:** Qian CHEN, Zhaoxu QIU, Yaobo ZHAO, Lu BAI, Yuhong CHEN, Sheng JIN, Fenfen MA, Jing DAI

**Affiliations:** 1 Department of Physiology, Hebei Medical University, Hebei, China; 2 Beijing Tiantan Hospital, China National Clinical Research Center for Neurological Diseases, Beijing, China; 3 Department of Critical Care Medicine, The Fourth Hospital of Hebei Medical University, Hebei, China; 4 Department of Pharmacy, Shanghai Pudong Hospital, Fudan University Pudong Medical Center, Shanghai, China; 5 Laboratory of Smart Drug Delivery, Ministry of Education, School of Pharmacy, Fudan University, Shanghai, China; 6 Department of Clinical Diagnostics, Hebei Medical University, Hebei, China

**Keywords:** Kidney aging, Trimethylamine-N-oxide, ZBP1, Necroptosis, DMB

## Abstract

The present study was aimed to investigate whether trimethylamine-N-oxide (TMAO) contributed to kidney aging by activating necroptosis. Male C57BL/6J mice were randomly divided into Control group (3 months old) and Old group (18 months old), compared to 3-month-old controls, 18-month-old male C57BL/6J mice showed significant increases in plasma creatinine (Cre) and blood urea nitrogen (BUN) (*P*<0.05), enhanced renal fibrosis (*P*<0.001), elevated plasma TMAO (*P*<0.01), and upregulation of senescence markers p53, p21, and p16 (*P*<0.05, *P*<0.01, and *P*<0.001, respectively). In order to investigate the effects of TMAO on kidney aging, the mice were intraperitoneally injected with TMAO for one to three months, mice showed time-dependent increases in Cre and BUN (*P*<0.05, respectively), progressive fibrosis, and gradual upregulation of senescence markers, ZBP1, and phosphorylation of RIPK3 and MLKL (*P*<0.05, respectively). In addition, three months of DMB treatment (inhibitor for TMAO formation) significantly reduced the plasma Cre and BUN levels (*P*<0.001 and *P*<0.05), downregulated the senescence markers expression, and improved kidney fibrosis (*P*<0.001 or *P*<0.05, respectively). In conclusion, our studies revealed that TMAO induced kidney aging by activating ZBP1-mediated necroptosis. Moreover, the inhibition of TMAO generation might be a potential treatment for kidney aging.

## Introduction

In the past several decades, due to advances in living conditions and health care, human life expectancy has increased dramatically while mortality has significantly decreased, followed by the progressively ageing population. Aging is defined as a time-dependent progressive decline of physiological functions, which represents the primary risk factor for chronic diseases and mortality [1]. As the main site of age-related changes, kidney aging grows to be nonnegligible issue [2–3]. Although structural and functional changes in kidney aging have been well documented, the detailed molecular mechanism responsible for these changes remains largely elusive. Recently, a growing body of evidence suggested that trimethylamine-N-oxide (TMAO), a gut microbiota-dependent metabolite, was closely associated with cellular aging and age-related diseases [4–5]. It was found that circulating level of TMAO was increased during the aging process and pharmacological inhibition of TMAO reversed endothelial dysfunction with aging [6]. Moreover, TMAO has been demonstrated to be not only a biomarker for kidney diseases, but also a contributor to the progression of kidney diseases [7–9]. However, whether TMAO contributes to kidney aging and the underlying mechanisms have not yet been fully investigated.

Even in the absence of age-related comorbidities, aging is significantly associated with progressive nephron loss and fibrosis, which is affected by programmed cell death (PCD) pathways, such as apoptosis, necroptosis, and pyroptosis [10–11]. Mounting evidence suggested that TMAO aggravated organ damage by activating some PCD pathways. For example, TMAO promoted hyperoxaluria-induced kidney injury by activating autophagy and apoptosis [12]. TMAO also enhanced the infiltration of M1 macrophages in atria, ultimately causing atrial structural remodeling through activating pyroptosis pathway [13]. To date, no experimental or clinical evidence has established a causal link between TMAO and necroptosis.

With this in mind, the aim of the present study was to investigate whether TMAO contributed to kidney aging by activating necroptosis pathway.

## Methods

### Animals and treatments

All male C57BL/6J mice were obtained from Vital River Laboratories (Beijing, China). Mice were housed in a standard environment at a temperature of 22–24 °C, 60 % humidity and 12 h light/12 h dark cycles and free access to water and chow diet. All animal experimental were performed according to the Guide for the Care and Use of Laboratory Animals published by the US National Institutes of Health (NIH Publication, 8th Edition, 2011) and approved by the Ethics Committee for Laboratory Animals Care and Use of Hebei Medical University.

In order to observe the effect of aging on kidney function, male C57BL/6J mice were randomly divided into 2 groups: Control group (3 months old) and Old group (18 months old).

In order to observe the effect of TMAO, mice at 8 weeks of age were randomly divided into 4 groups: Control group, TMAO-1m group, TMAO-2m group, and TMAO-3m group. The mice in the TMAO-1m group, TMAO-2m group, and TMAO-3m group were intraperitoneally injected with TMAO (100 μmol/kg/day, Aladdin Biochemical Technology Co., Ltd., China) for one, two, and three months, respectively, whereas the mice in the Control group were injected with normal saline.

In order to observe the effect of 3,3-dimethyl-1-butanol (DMB, the TMA lyase inhibitors) on kidney aging, male C57BL/6J mice at 15 months of age were randomly divided into 2 groups: Old group and Old + DMB. The mice in the Old + DMB group were fed with 1.3 % DMB (Aladdin Biochemical Technology Co., Ltd., Shanghai, China) in the drinking water for 3 months, while the mice in the Old group were fed with normal drinking water for the same period.

At the end of the experiment, the mice were euthanized by intraperitoneally injecting an overdose of pentobarbital (100 mg/kg). After blood was collected from abdominal aorta, plasma was separated from the blood by centrifuged at 12000× g for 15 min and stored at −80 °C until assay. Subsequently, the kidney tissue samples were rapidly removed and fixed with 4 % paraformaldehyde or frozen at −80 °C until further analysis.

### Measurement of creatinine (Cre) and blood urea nitrogen (BUN) concentration in plasma

The plasma levels of Cre and BUN were determined with the corresponding assay kits (Jiancheng Bioengineering Institute, China) according to the manufacturer’s instructions.

### Measurement of TMAO concentration in plasma

The plasma TMAO levels were measured by liquid chromatography with mass spectrometry according to the previously study [14].

### Masson’s trichrome analysis

After fixed in 4 % paraformaldehyde for 48 h, the kidney tissues were dehydrated, permeabilizated, embedded in paraffin, sectioned at 5-μm thickness, and stained with Masson’s trichrome to identify collagen deposition, which was shown in blue. The kidney sections were examined using an optical microscope (Olympus, Japan) and the collagen volume fraction was calculated as the percentage of collagen (blue-stained area) to the total renal area.

### Western blot analysis

The frozen kidney tissues were homogenized with ice-cold radio immunoprecipitation assay (RIPA) lysis buffer (Beyotime Biotechnology, China). After centrifugation (12000× g) at 4 °C for 20 min, the supernatant was collected and the protein concentration quantified by using a bicinchoninic acid (BCA) Protein Assay Kit (Beyotime Biotechnology, China) according to the manufacturer’s instructions. Equal amounts of protein samples were separated on the sodium dodecyl sulfate – polyacrylamide gel electrophoresis gels and then transferred onto polyvinylidene difluoride membranes. The membranes were blocked with 5 % non-fat milk for 1 h and incubated with primary antibodies that recognized Z-DNA binding protein 1 (ZBP1, 1:500, Proteintech Biotechnology, USA), mixed-lineage kinase domain-like pseudokinase (MLKL, 1:2000, Proteintech Biotechnology, USA), phosphorylation of MLKL (p-MLKL, 1:1000, Abcam, USA), receptor-interacting protein kinase 3 (RIPK3, 1:1000, Proteintech Biotechnology, USA), phosphorylation of RIP3 (p-RIPK3, 1:1000, Abcam, USA), cleaved Caspase-3 (1:1000, Proteintech Biotechnology, USA), cleaved Caspase-8 (1:500, Proteintech Biotechnology, USA), NOD-like receptor protein 3 (NLRP3, 1:1000, Proteintech Biotechnology, USA), interleukin-1β (IL-1β, 1:1000, Proteintech Biotechnology, USA), p53 (1:1000, Proteintech Biotechnology, USA), p21 (1:1000, HUABIO, China), p16 (1:1000, HUABIO, China), β-Tubulin (1:5000, Wanleibio, China), and GAPDH (1:5000, Proteintech Biotechnology, USA) at 4 °C overnight. Then the membranes were incubated with horseradish peroxidase-conjugated secondary antibodies for 1 h after washing with TBST. The specific bands were visualized using an ultrasensitive chemiluminescent solution and analyzed on a gray scale using ImageJ software (National Institutes of Health, USA).

### Statistical analysis

Results were presented as mean ± SEM and statistical analysis was performed using SPSS software package (SPSS 17.0, Inc., USA). Differences between two groups were assessed using an independent *t*-test, while differences among more than two groups were analyzed by one-way ANOVA followed by *post hoc* Dunnett’s tests. *P*<0.05 was considered statistically significant.

## Results

### Aging led to kidney dysfunction and increased TMAO levels

**Fig. 1:**
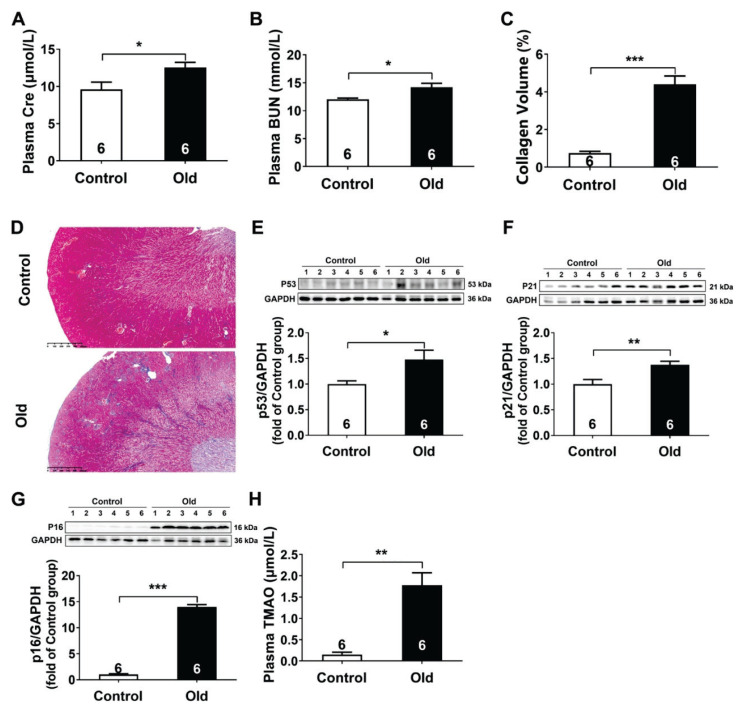
Aging led to kidney dysfunction and increased TMAO levels. (**A**) Creatinine (Cre) levels in the plasma (n=6). (**B**) Blood urea nitrogen (BUN) levels in the plasma (n=6). (**C**) The quantitative analysis for collagen volume fraction (%) in kidney tissues (n=6). (**D**) Representative Masson’s trichrome-stained kidney sections (n=6). (**E–G**) Representative western blots and quantitative analysis for p53, p21, and p16 protein expression in kidney tissues (n=6). (**H**) TMAO levels in the plasma (n=6). Results are expressed as mean ± SEM. * *P*<0.05, ** *P*<0.01, *** *P*<0.001.

As shown in Fig. 1A–B, the aged mice exhibited marked kidney dysfunction, characterized by 30.87 % and 18.05 % increases in plasma Cre and BUN levels, the two indicators of kidney functions, respectively. Masson staining demonstrated an obvious larger area of fibrosis than that in the Control group (4.40 % vs. 0.75 %) (Fig. 1C–D). Further western blot analyses showed significant upregulation of senescence-associated markers, including p53 (1.5-fold), p21 (1.4-fold), and p16 (14-fold) in the aged mice (Fig. 1E–G). Notably, plasma TMAO levels were also significantly increased in the aged mice (Fig. 1H).

### ZBP1-mediated necroptosis was activated in aging kidney

**Fig. 2:**
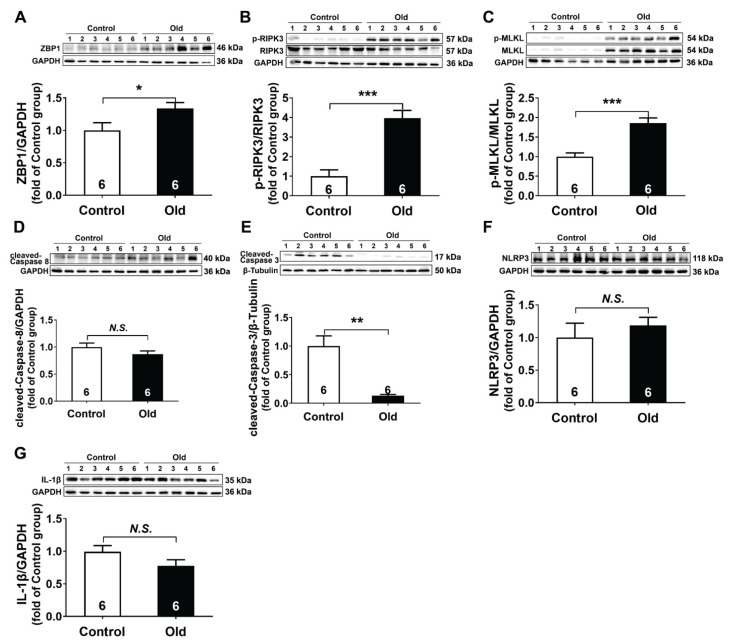
ZBP1-mediated necroptosis was activated in aging kidney. (**A–G**) Representative western blots and quantitative analysis for ZBP1, p-RIP3/RIP3, p-MLKL/MLKL, cleaved-Caspase 8, cleaved-Caspase 3, NLRP3, and IL-1β protein expression in kidney tissues (n=6). Results are expressed as mean ± SEM. * *P*<0.05, ** *P*<0.01, *** *P*<0.001.

Subsequently, we examined the PCD pathways, such as necroptosis, apoptosis, and pyroptosis in the aging kidney. The results showed that the expression of ZBP1 (1.3-fold), p-RIPK3/RIPK3 ratio (4-fold) and p-MLKL/MLKL ratio (1.9-fold) were significantly increased in the Old group as compared with the Control group (Fig. 2A–C). However, cleaved-caspase 3 was downregulated and cleaved-caspase 8, NLRP3 and IL-1β were not significantly different between the two groups (Fig. 2D–G). These results indicated that the ZBP1-mediated necroptosis was activated in aging kidney.

### TMAO induced age-related kidney dysfunction by activating necroptosis

**Fig. 3:**
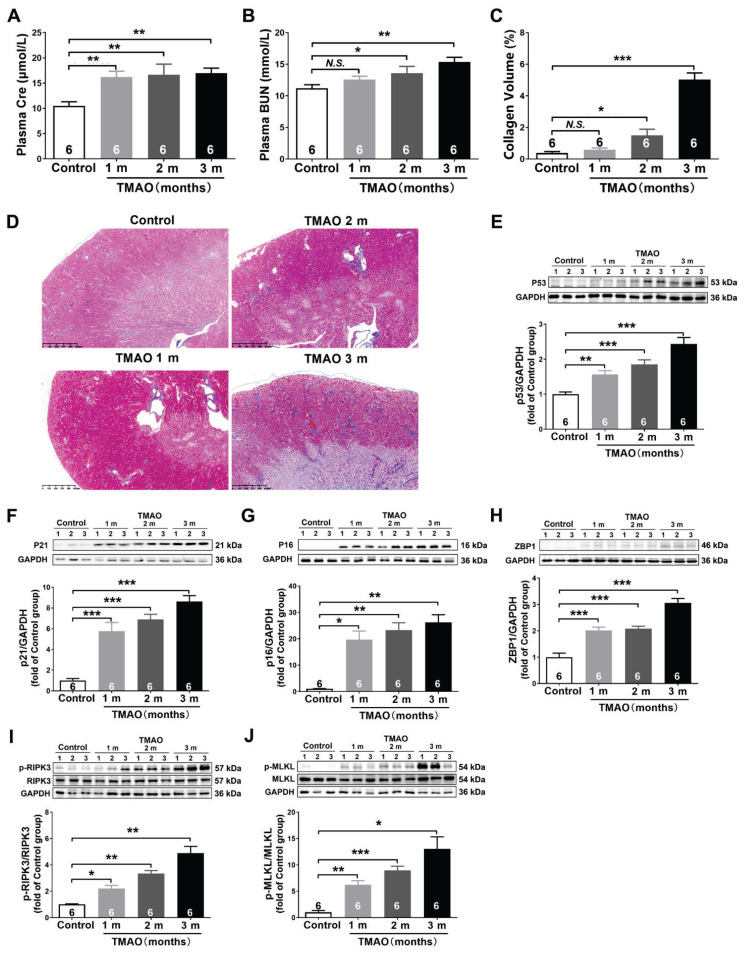
TMAO induced age-related kidney dysfunction by activating necroptosis. (**A**) Creatinine (Cre) levels in the plasma (n=6). (**B**) Blood urea nitrogen (BUN) levels in the plasma (n=6). (**C**) The quantitative analysis for collagen volume fraction (%) in kidney tissues (n=6). (**D**) Representative Masson’s trichrome-stained kidney sections (n=6). (**E–J**) Representative western blots and quantitative analysis for p53, p21, p16, ZBP1, p-RIP3/RIP3, and p-MLKL/MLKL protein expression in kidney tissues (n=6). Results are expressed as mean ± SEM. * *P*<0.05, ** *P*<0.01, *** *P*<0.001.

In order to investigate the effects of TMAO on kidney aging, the mice were intraperitoneally injected with TMAO for one, two, or three months, respectively. With the extension of TMAO treatment time, the plasma Cre and BUN levels were increased gradually (Fig. 3A–B), accompanied by the progressive kidney fibrosis (Fig. 3C–D). Analysis of senescence markers showed a time-dependent upregulation of p53, p21 and p16 (Fig. 3E–G). In addition, the ZBP1, phosphorylation ratios of RIPK3 (p-RIPK3/RIPK3) and MLKL (p-MLKL/MLKL) expression were progressively upregulated (Fig. 3H–J). These findings suggested that TMAO induced age-related kidney dysfunction by activating ZBP1-mediated necroptosis.

### DMB alleviated age-related kidney dysfunction

**Fig. 4:**
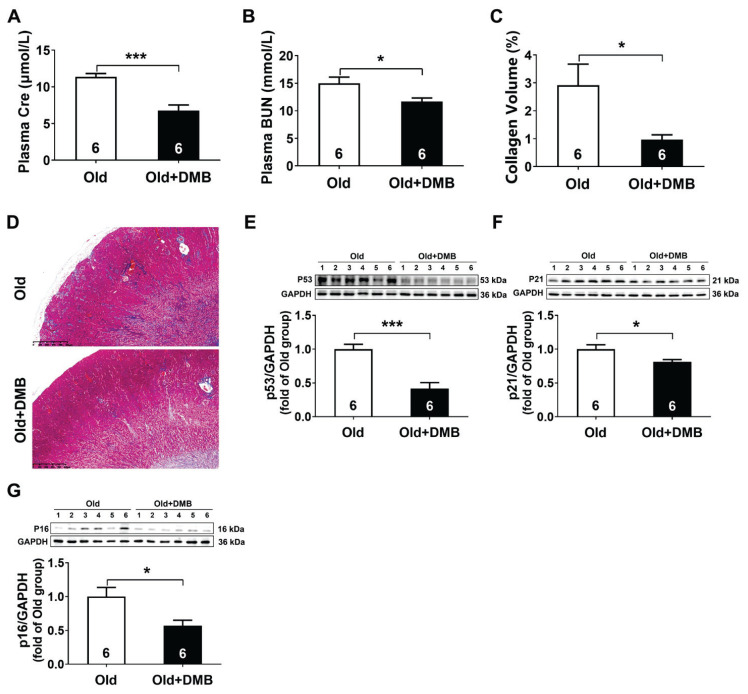
DMB alleviated age-related kidney dysfunction. (**A**) Creatinine (Cre) levels in the plasma (n=6). (**B**) Blood urea nitrogen (BUN) levels in the plasma (n=6). (**C**) The quantitative analysis for collagen volume fraction (%) in kidney tissues (n=6). (**D**) Representative Masson’s trichrome-stained kidney sections (n=6). (**E–G**) Representative western blots and quantitative analysis for p53, p21, and p16 protein expression in kidney tissues (n=6). Results are expressed as mean ± SEM. * *P*<0.05, ** *P*<0.01, *** *P*<0.001.

To further confirm that TMAO induced kidney aging, DMB was used in aged mice for 3 months to inhibit TMAO formation. The results showed that the plasma Cre and BUN levels significantly decreased by approximately 40.30 % and 22.08 % respectively (Fig. 4A–B), accompanied by a 55.64 % decrease in kidney fibrosis (Fig. 4C–D) after DMB treatment. Western blot analysis revealed a pronounced downregulation of the age-related proteins p53, p21 and p16 after DMB treatment, with the expression decreasing by approximately 58.33 %, 18.96 %, and 43.01 % respectively compared to the Old group (Fig. 4E–G). These results suggested that inhibition of TMAO production by DMB alleviated age-related kidney dysfunction.

## Discussion

In the present study, we found that plasma TMAO levels were increased in the aged mice and intraperitoneal injection of TMAO time-dependently induced age-related kidney dysfunction by activating ZBP1-mediated necroptosis, whereas the inhibition of TMAO generation reversed the kidney dysfunction in the aged mice.

TMAO is a gut microbiota-derived metabolite produced primarily from dietary choline, L-carnitine, and betaine. Gut microbiota converts these substances to generate trimethylamine (TMA), an intermediate compound. Subsequently, the gaseous TMA is rapidly absorbed into the circulation and oxidized into TMAO by hepatic flavin-containing monooxygenases (FMO). TMAO is then either transported to the tissues or mainly cleared unchanged by the kidney. Thus, the circulating concentrations of TMAO are negatively correlated with kidney function [15–16]. As one of the organs whose function declines most rapidly with aging, the structural and functional changes occur in the aging kidneys, including reduction of nephron number and fibrosis, which reduce TMAO clearance from kidney, resulting in the accumulation of circulating TMAO. In turn, the accumulative TMAO further exacerbates the aging-related dysfunction of multiple organs, including the kidney [5,17]. In the present study, we found that plasma Cre and BUN levels, the indicators of kidney function, were significantly increased in the 18-month-old mice, and Masson staining assay also showed a larger area of fibrosis than that in the 3-month-old mice. In addition, the plasma TMAO levels were significantly elevated in the 18-month-old mice. Whereas, three months of DMB treatment (inhibitor for TMAO formation through inhibition of microbial TMA lyases) significantly reduced the plasma Cre and BUN levels and improved kidney fibrosis. In order to observe the effect of TMAO on kidney aging, mice at 8 weeks of age were intraperitoneally injected with TMAO for one, two, or three months, respectively. The results showed that kidney fibrosis and kidney injury were aggravated as the extension of TMAO treatment time. Meanwhile the expression of important aging markers, including p53, p21, and p16, were gradually upregulated in the kidney, which indicated that TMAO promoted kidney aging. Previous studies have suggested that TMAO played important roles in chronic kidney disease [18], and there was a lack of research on kidney aging, but the studies about TMAO promoting aging in other organs were consistent with our results. It was reported that TMAO accelerated endothelial cell senescence and vascular aging [19], whereas initiation of DMB at midlife prevented endothelial dysfunction and attenuated aortic stiffening with ageing in mice [20]. Aging also increased TMAO levels in the brain, and TMAO contributed to neuroinflammatory and astrocyte-mediated modulation of cognitive performance with normal aging [21].

PCD pathways, such as apoptosis, necroptosis, and pyroptosis, are essential for maintaining normal cell turnover and tissue homeostasis. However, the dysregulated PCD contributes aging and aging-related disease [10,22]. For example, impaired apoptosis permitted the survival of senescent cells, leading to aging [23]. Chronic sterile inflammation induced by necroptosis or pyroptosis activation, the two typical inflammatory cell death, was also the primary contributor to aging [24,25]. In the present study, we found that ZBP1, p-RIPK3/RIPK3 ratio and p-MLKL/MLKL ratio were significantly increased in the Old group as compared to the Control group, while cleaved-caspase 3 was downregulated and cleaved-caspase 8, NLRP3 and IL-1β were not significantly different between the two groups. RIPK3 and MLKL are the key players in the necroptosis signaling pathway. After being activated by ZBP1, RIPK3 phosphorylates itself and thereby become activated to phosphorylate MLKL which finally executes necroptosis [26,27]. These results indicated that the ZBP1-mediated necroptosis was activated in aging kidney, but apoptosis and pyroptosis were not involved in. Although current studies directly linking necroptosis to kidney aging remained scarce, the existing studies in liver and brain were line with our findings [28,29]. In addition, accumulated evidence showed that necroptosis was the main pathophysiological mechanism of nephron loss and kidney fibrosis [30]. Then we confirmed whether TMAO activated necroptosis during kidney aging and found that the ZBP1, phosphorylation ratios of p-RIPK3/RIPK3 and p-MLKL/MLKL were progressively upregulated with the extension of TMAO treatment time, which suggested that TMAO induced the activation of necroptosis. It was reported that TMAO exacerbated mitochondrial dysfunction and increased mitochondrial ROS, leading to mitochondrial DNA (mtDNA) leakage into the cytosol [31]. This cytosolic mtDNA directly engaged ZBP1 and initiated its oligomerization, which recruited and activated RIPK3 [32]. However, there was no direct evidence to confirm how TMAO activated ZBP1 and the precise molecular mechanisms involved remain elusive, necessitating further exploration.

Several limitations of the present study should be acknowledged. First, this study only revealed a causal relationship between kidney aging and necroptosis activation without fully excluding alternative pathways. Other cell death pathways, such as autophagy, copper hyperplasia and parthanatos, should be considered to fully describe the role of TMAO in kidney aging. In addition, the application of specific inhibitors of necroptosis may help to determine that necroptosis is the sole performer of this process. Second, although the selection of 3-month (young) and 18-month (aged) mice effectively represents the two ends of the aging spectrum, the addition of intermediate age groups (e.g., 12-month-old) or older age groups (e.g., 24-month-old) will help to delineate the timeline of TMAO accumulation and pathway activation. Third, although DMB is widely used to inhibit TMAO production by targeting gut microbial TMA lyases, we cannot fully exclude potential off-target effects that might contribute to the observed outcomes.

In conclusion, our study studies revealed that TMAO induced kidney aging by activating ZBP1-mediated necroptosis. Moreover, the inhibition of TMAO generation might be a potential treatment for kidney aging.
